# Effects of Acetamiprid at Low and Median Lethal Concentrations on the Development and Reproduction of the Soybean Aphid *Aphis glycines*

**DOI:** 10.3390/insects13010087

**Published:** 2022-01-13

**Authors:** Aonan Zhang, Ling Xu, Ziqi Liu, Jiabo Zhang, Kuijun Zhao, Lanlan Han

**Affiliations:** College of Agriculture, Northeast Agricultural University, Harbin 150030, China; zan826319@163.com (A.Z.); 18846918117@163.com (L.X.); zan8261314@163.com (Z.L.); zhangjiabo_1020@163.com (J.Z.); kjzhao@163.com (K.Z.)

**Keywords:** neonicotinoid, hormesis, life table, survival rate, population

## Abstract

**Simple Summary:**

We conducted a study on the effects of LC_50_ and LC_30_ acetamiprid on the growth and development of the soybean aphid *Aphis glycines* Matsumura (Hemiptera: Aphididae). We found that exposure to acetamiprid at LC_50_ significantly extended the mean generation time, adult pre-ovipositional period, and total pre-reproduction period compared to the control, whereas exposure to acetamiprid at LC_30_ significantly shortened these periods. Acetamiprid at LC_50_ treatment significantly decreased the growth rate compared with the LC_30_ treatment. In addition, this study also found that low lethal concentration of acetamiprid could advance the occurrence of a reproductive peak, which could help us understand the major occurrence period of soybean aphids under acetamiprid stress. The present study provides reference data that could facilitate the exploration of the effects of acetamiprid on *A. glycines* in the field.

**Abstract:**

The soybean aphid *Aphis glycines* Matsumura (Hemiptera: Aphididae) is a major pest of soybean and poses a serious threat to soybean production. Studies on the effect of acetamiprid on the life table parameters of *A. glycines*, provide important information for the effective management of this pest. We found that exposure to acetamiprid at LC_50_ significantly extended the mean generation time, adult pre-reproductive period, and total pre-reproduction period compared with the control, whereas exposure to acetamiprid at LC_30_ significantly shortened these periods. Exposure to acetamiprid at both LC_30_ and LC_50_ significantly decreased the fecundity of the female adult, net reproductive rate, intrinsic rate of increase, and finite rate of increase compared with the control. The probability of attaining the adult stage was 0.51, 0.38, and 0.86 for a newly born nymph from the LC_30_ acetamiprid treatment group, LC_50_ acetamiprid treatment group, and control group, respectively. Acetamiprid at both LC_50_ and LC_30_ exerted stress effects on *A. glycines*, with the LC_50_ treatment significantly decreased the growth rate compared with the LC_30_ treatment. The present study provides reference data that could facilitate the exploration of the effects of acetamiprid on *A. glycines* in the field.

## 1. Introduction

The soybean aphid, *Aphis glycines* Matsumura (Hemiptera: Aphididae), was first found in North America in 2000, and thereafter rapidly spread across most of North America, including the north–central and northeastern United States and southeastern Canada. In Asia, *A. glycines* does not usually erupt in large numbers, but in North America, it has decreased yields and its control incurs high costs [1]. Severe infestation by these aphids can result in a 50–80% reduction in soybean production [2,3].

Acetamiprid, a neonicotinoid, has been extensively used against soybean aphids on crops, such as soybeans, cotton, tobacco, potatoes, tomatoes, and nuts [4,5,6]. However, this pesticide also has some negative effects, particularly physiological and behavioral hormesis effects associated with low or sublethal concentrations, due to frequent use [7]. Low or sublethal concentrations of acetamiprid in the field are mainly a result of its continuous degradation by plants, animals, fungi, and bacteria [8,9]. For example, low doses of acetamiprid promote the development of resistance against it in the progeny of the melon aphid, *Aphis gossypii* Glover [10]. In recent years, reports on aphid reproduction and development, and resistance development to acetamiprid at low lethal concentrations, have been increasing. For example, adult longevity and total longevity were significantly shortened, and fecundity decreased significantly when *Brevicoryne brassicae* L. (Homoptera: Aphididae) was exposed to acetamiprid at the LC_30_ [11]. In addition, Ullah et al. demonstrated that the mean developmental times of the first, third, and fourth instars of *A. gossypii* in acetamiprid-resistant populations were significantly shorter than those of sensitive populations [12].

Acetamiprid at low or sublethal concentrations have transgenerational effects on insect pests [13,14]. Ullah et al., observed such a phenomenon when they treated *A. gossypii* with acetamiprid at the LC_15_ concentration. The population of the F0 generation was reduced significantly, whereas the pre-adult developmental period of the F1 generation was significantly prolonged. Moreover, acetamiprid at the LC_15_ concentration upregulated the expression of the *Vg* gene in the *A. gossypii* progeny. These results indicated a link between acetamiprid and the stimulation of reproduction in the F1 generation [15]. Furthermore, P450 genes, especially *CYP6CY14*, *CYP6DC1*, *CYP6CZ1*, and *CYP6DD1*, were hypothesized to be potentially involved in acetamiprid resistance in *A. gossypii* [10]. In addition, insecticides administered at low or sublethal concentrations could lead to the rapid deterioration of the ecological environment and exert stress on the natural enemies of the target insects and other beneficial insects [16,17,18].

Consequently, it is essential to investigate the population dynamics of soybean aphids under insecticide stress, and actively explore the relationship among insecticides, pests, and the environment [19,20]. Therefore, we explored survival, reproduction, development, longevity, and fertility trends and data related to individual differences and developmental stages based on the age-stage, two-sex life table [21]. This provided a better assessment of stage-specific mortality [22,23]. Based on the age-stage, two-sex life table of *A. glycines*, we evaluated the effects of acetamiprid at low lethal concentrations on reproduction and development. The present study serves as a reference for the rational and effective use of acetamiprid in the control of *A. glycines* populations.

## 2. Materials and Methods

### 2.1. Laboratory Aphid Population and Chemical Agents

The laboratory strain of *A. glycines* used in this study was originally collected from a soybean field in Xiangyang farm, Harbin, Heilongjiang, China. The population was cultured separately in the laboratory of Northeast Agricultural University for many years and was never exposed to any insecticides. Dongnong 52 soybean plants were used to maintain the strain of *A. glycines*. Soybean plants were maintained at 25 ± 1 °C, a relative humidity of 65–70%, and a photoperiod of 14:10 (L:D) h. A large tray could hold twelve pots with soybeans. Each large tray was placed in an insect proof cage, which prevented contamination of the colony from other aphids. Four new aphid-free pots were added to the colony weekly. This provided a homogeneous soybean plant for the aphids to feed on [24].

Water-dispersible granules of the insecticide (70% acetamiprid, commercial name: Jueci) was purchased from Jiangxi Jinnong Chemical Co., Ltd. (Yichun, Jiangxi, China). Calcium nitrate, potassium nitrate, potassium dihydrogen phosphate, magnesium sulfate, disodium ethylenediaminetetraacetic acid (disodium EDTA), and streptomycin sulfate were purchased from Shanghai Alighting Biochemical Technology Co., Ltd. (Shanghai, China).

### 2.2. Preparation of Culture Medium

*Transparent* plastic Petri dishes (6 cm diameter × 1.5 cm height) were used to perform bioassay with the first instar A. glycines nymphs. The ratio components of concentrated plant nutrient solution were as follows: calcium nitrate (4.1 g), potassium nitrate (2.5 g), potassium dihydrogen phosphate (0.7 g), magnesium sulfate (0.6 g), 1.54% disodium EDTA aqueous solution (5.0 mL), one million units of streptomycin sulfate (0.05 g), and distilled water (5.0 L). The diluent was obtained by mixing the plant nutrient solution concentrate with distilled water at a ratio of 1:3. Agar was prepared by mixing 1% *w*/*w* agar powder with diluent and then it was boiled. After cooling for 10 min, the agar was poured into the Petri dishes. The leaf disc was obtained by cutting the leaves with a metal tube. Each disc was attached to the surface of the medium. We gently touched each soybean aphid with a small wet brush which caused it moving on the leaf disc. After that, we gently lifted the soybean aphid again with a small brush and transferred it to the newly attached leaf disc. To keep the soybean aphid in its natural feeding state, we turned each Petri dish upside down.

### 2.3. Selection of Acetamiprid Concentration

Acetamiprid is frequently used by farmers in the field [25]. Therefore, the LC_50_ used in this study was based on that as applied in the field. Moreover, concentrations exceeding LC_50_ would be detrimental to the aphid population in the laboratory and therefore would affect the establishment of the life table. Similarly, the LC_30_ used in this study was a low lethal concentration to which the soybean aphid may be exposed after acetamiprid degradation in the field, which could affect its growth and development. At the same time, preliminary experiments showed that acetamiprid at sublethal concentration had no significant effect on soybean aphids. Therefore, we chose these LC_50_ and LC_30_ acetamiprid to stress the soybean aphids, and monitored the effects on their growth and development.

### 2.4. Dose–Response Bioassay

A dose–response bioassay was conducted with the first instar *A. glycines* nymphs using the leaf dip method recommended by the Insecticide Resistance Action Committee. Acetamiprid was diluted with 1% acetone and 0.05% (*v*/*v*) Triton X-100. Seven concentrations of acetamiprid were prepared (30.25, 25.95, 16.45, 8.90, 5.23, 3.40, and 2.30 mg a.i./L). Each leaf disc was immersed for 10 s, independently, in each solution, and then placed on paper to dry. The control leaf disc was immersed in distilled water which contained 1% acetone and 0.05% (*v*/*v*) Triton X-100. The air-dried discs were attached to the agar medium with the top-side facing down, and the first instar nymphs were placed on them. Sixty first instar nymphs were used for dose–response bioassays at each concentration; there were three replicates per concentration, and each replicate contained 20 nymphs. Mortality was determined 24 h after exposure. The nymphs were considered dead if they were found standing on their heads, or not moving when stimulated with a small brush [12]. The toxicity of acetamiprid to nymphs was statistically analyzed using the concentration–mortality regression line and a log-probit model of SPSS (version 23.0, SPSS Inc., Chicago, IL, USA), and the LC_50_ and LC_30_ values were obtained.

### 2.5. Life–History Study

Eighty adults were equally divided among eight leaf discs. Each leaf disc was kept in a separate Petri dish. The newly first instar nymphs were selected 24 h later and transferred to a leaf disc which was pre-impregnated with acetamiprid or distilled water. After 24 h, the old leaf discs in each Petri dish were replaced with new ones free of acetamiprid. After that, fresh leaf discs were replaced every 24 h. One hundred first instar nymphs were exposed to each treatment; each first instar nymph was taken as one replicate. The growth, survival, mortality, and fecundity of the individuals were observed until all organisms died.

### 2.6. Reproductive Parameter Calculation

The age-stage-specific survival rate (*s_xj_*, *x* = age, *j* = stage), age-specific survival rate (*l_x_*), age-stage specific fecundity (*f_xj_*), and age-specific fecundity (*m_x_*) were calculated as follows [1,22]:

(1) $$s_{xj}=\frac{n_{xj}}{n_{01}}$$

(2) $$l_{x}=\sum_{j=1}^{k}s_{xj}$$

(3) $$m_{x}=\frac{\sum_{j=1}^{k}s_{xj}f_{xj}}{\sum_{j=1}^{k}s_{xj}},$$

where, *n*_01_ stands for the number of the first instar nymphs, and *k* stands for the number of stages. The intrinsic rate of increase (*r*), finite rate of increase (*λ*), mean generation time (*T*), and net reproductive rate (*R*_0_) were calculated as follows [26]:

(4) $$\sum_{x=0}^{\infty }e^{-r(x+1)}l_{x}m_{x}=1,$$

(5) $$\lambda =e^{r},$$

(6) $$T=\frac{\ln\;R_{0}}{r},$$

(7) $$R_{0}=\sum_{x=0}^{\infty }l_{x}m_{x}.\;$$

The life expectancy (*e_xj_*), that is, the time that an individual of age *x* and stage *j* is expected to live, was calculated according to [27] as:

(8) $$e_{xj}=\sum_{i=x}^{\infty }\sum_{y=j}^{k}{s^{\prime }}_{iy},\;$$

where, *s′_iy_* is the probability that an individual of age *x* and stage *j* would survive to age *i* and stage *y.* Tuan [28] defined the reproductive value (*v_xj_*) as follows:

(9) $$v_{xj}=\frac{e^{r(x+1)}}{s_{xj}}\sum_{i=x}^{\infty }e^{-r(i+1)}\sum_{y=j}^{k}{s^{\prime }}_{iy}f_{iy}$$

The mean values and standard errors of the population parameters, mean longevity of the first to fourth instar nymphs and adults, adult and total pre-reproductive period, and mean fecundity were analyzed using the TWOSEX-MSChart software (2020 version). Paired bootstrap test (*B* = 100,000) [29], which was based on the percentile of differences and 95% CI of normalized distribution of differences, was used to compare the differences among treatments. All curve graphs were generated using SigmaPlot 12.0.

## 3. Results

### 3.1. Dose–Response Bioassay with the First Instar A. glycines Nymphs

The toxicity of acetamiprid against the first instar *A. glycines* nymphs was investigated (Table 1). The LC_50_ value of acetamiprid was estimated as 6.742 mg a.i./L with a confidence interval of 5.133–8.629 mg a.i./L, and the LC_30_ value was estimated as 3.968 mg a.i./L with a confidence interval of 2.676–5.203 mg a.i./L, respectively.

### 3.2. Life–History Traits

Acetamiprid affected the developmental time, longevity, and fecundity of *A. glycines* (Table 2). Compared with the control group, exposure to acetamiprid at LC_50_ increased the developmental time of the first instars (F = 79.499, df = 120.089, *p* = 0.000), the adult pre-reproductive period (APOP, F = 67.926, df = 136.213, *p* = 0.000), and the total pre-reproductive period (TPOP, F = 100.120, df = 108.570, *p* = 0.000), but decreased fecundity (F = 33.969, df = 147.220, *p* = 0.000) and adult longevity (F = 129.689, df = 101.378, *p* = 0.000). When exposed to acetamiprid at LC_30_, the APOP (F = 92.152, df = 114.331, *p* = 0.000), TPOP (F = 63.304, df = 129.309, *p* = 0.000), and fecundity (F = 87.438, df = 117.607, *p* = 0.000) of *A. glycines* decreased significantly. 

### 3.3. Life Table and Fertility Parameters

The *r* was significantly lower in the LC_50_ acetamiprid group (F = 6.161, df = 172.251, *p* = 0.000) than in the control group (Table 3). Acetamiprid at LC_30_ had a similar effect on the *r* (F = 10.404, df = 180.504, *p* = 0.000). The *T* of the LC_50_ acetamiprid group (F = 168.583, df = 101.018, *p* = 0.000) was longer than that of the control group. By contrast, the *T* of the LC_30_ acetamiprid group (F = 166.696, df = 101.178, *p* = 0.000) was shorter than that of the control.

**Figure 1:**
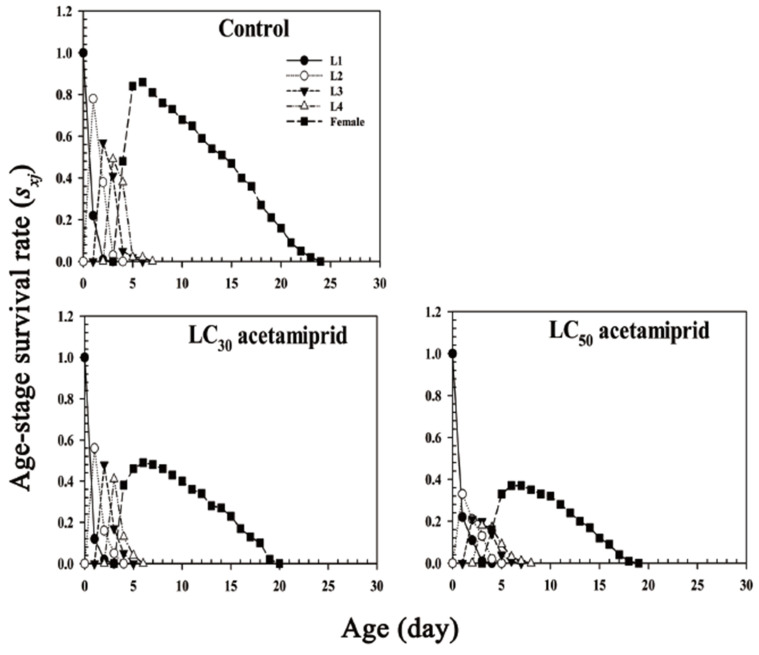
Age-stage specific survival rate (*s_xj_*) of *Aphis glycines*.

Age-stage survival rate curves (*s_xj_*) show the probability that a newborn nymph will survive to age *x* and stage *j* (Figure 1). Owing to the different development rates between individuals, the age-stage specific survival rate curves showed evident overlaps. The probability of attaining the adult stage was 0.51, 0.38, and 0.86 for a newly born nymph from the LC_30_ acetamiprid treatment group, LC_50_ acetamiprid treatment group, and control group, respectively. The adults’ peak was on the sixth day under the LC30 acetamiprid treatment and on both the sixth and seventh day under the LC50 acetamiprid treatment (The peak values on the 6th and 7th day were the same). The adult survival rate in both LC30 and LC50 acetamiprid treatment groups was lower than that in the control group. Meanwhile, the longest total longevity and adult longevity were recorded in the control group. The last individual of the LC30 acetamiprid treatment group, LC50 acetamiprid treatment group, and control group died on days 20, 19, and 24, respectively (Figure 1). 

The *s_xj_* of *Aphis glycines* were exposed to the following treatments: control, acetamiprid LC_30_, and acetamiprid LC_50_. L1, L2, L3, and L4 represent *s_xj_* of the first, second, third, and fourth instar nymphs, respectively.

**Figure 2:**
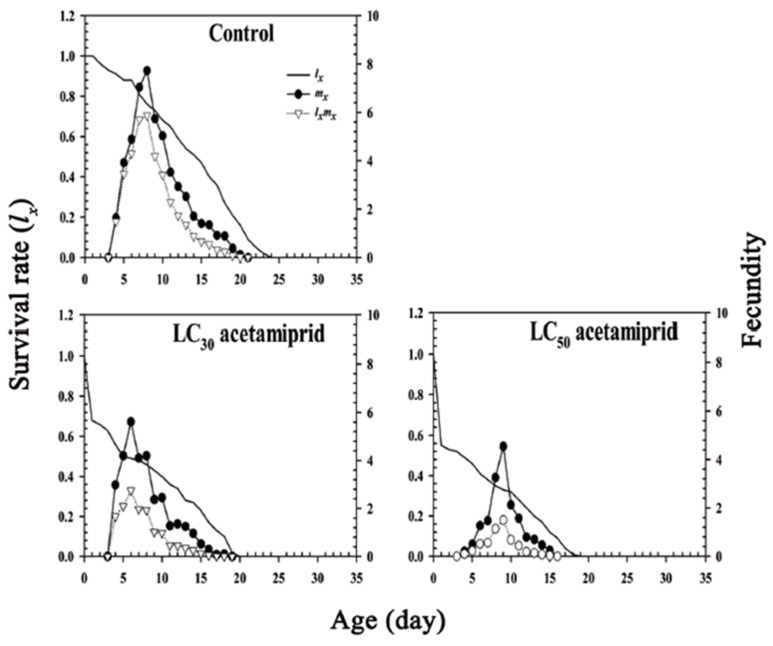
Age-specific survival rate (*l_x_*), age-specific fecundity of the total population (*m_x_*), and age-specific maternity (*l_x_m_x_*) of *Aphis* *glycines*.

The age-specific survival rate (*l_x_*) curve illustrates the likelihood that a newly-born nymph will survive to age *x* with no emphasis on nymphal development and stage differentiation (Figure 2). After acetamiprid treatment, the *l_x_* curve significantly decreased. The age-specific fecundity (*m_x_*) and age-specific maternity (*l_x_m_x_*) peaks of the adults in the control group were higher than those in the acetamiprid treatment group. It indicated that the stress of acetamiprid reduced the reproductive capacity of the soybean aphid population. The *m_x_* peak in the LC_30_ acetamiprid treatment group was on day 6, whereas that in the control group was on day 8. The *m_x_* peak in the LC_50_ acetamiprid group was on day 9, that is, 1 day later than that in the control group (Figure 2). The maximum age-specific maternity values were on days 8, 9, and 6 for the control, LC_50_ acetamiprid treatment, and LC_30_ acetamiprid treatment groups, respectively. 

The *l_x_*, *m_x_*, and *l_x_m_x_* of *Aphis* *glycines* were exposed to the following treatments: control, acetamiprid LC_30_, and acetamiprid LC_50_.

**Figure 3:**
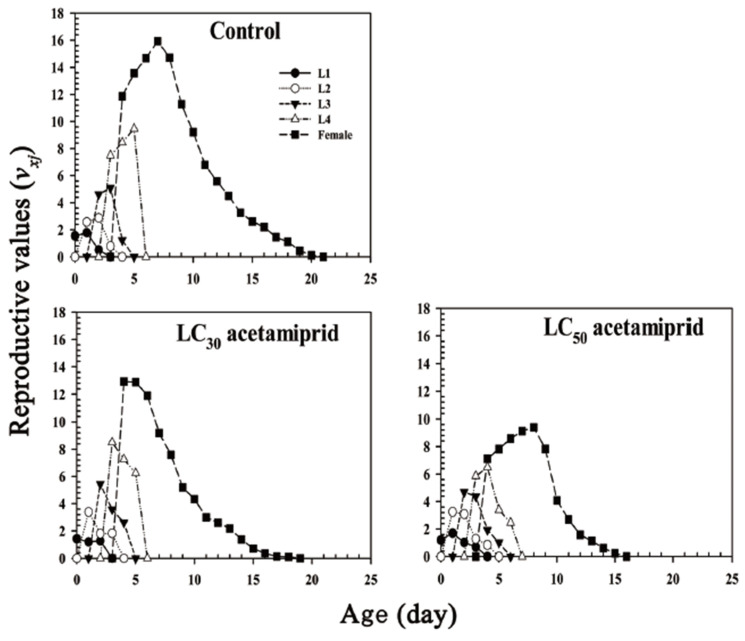
Age-stage specific reproductive values (*v_xj_*) of *Aphis* *glycines*.

Exposure to acetamiprid at different concentrations affected the age-stage-specific reproductive value (*v_xj_*) of *A. glycines* (Figure 3). A higher maximum reproductive value in each stage of *A. glycines* was observed in the control group than in the treatment groups. The earliest and latest reproductive value peaks were observed in the LC_30_ and LC_50_ acetamiprid treatment groups, respectively (Figure 3).

The *v_xj_* of *Aphis* *glycines* were exposed to the following treatments: control, acetamiprid LC_30_, and acetamiprid LC_50_. L1, L2, L3, and L4 represent *v_xj_* of the first, second, third, and fourth instar nymphs, respectively.

**Figure 4:**
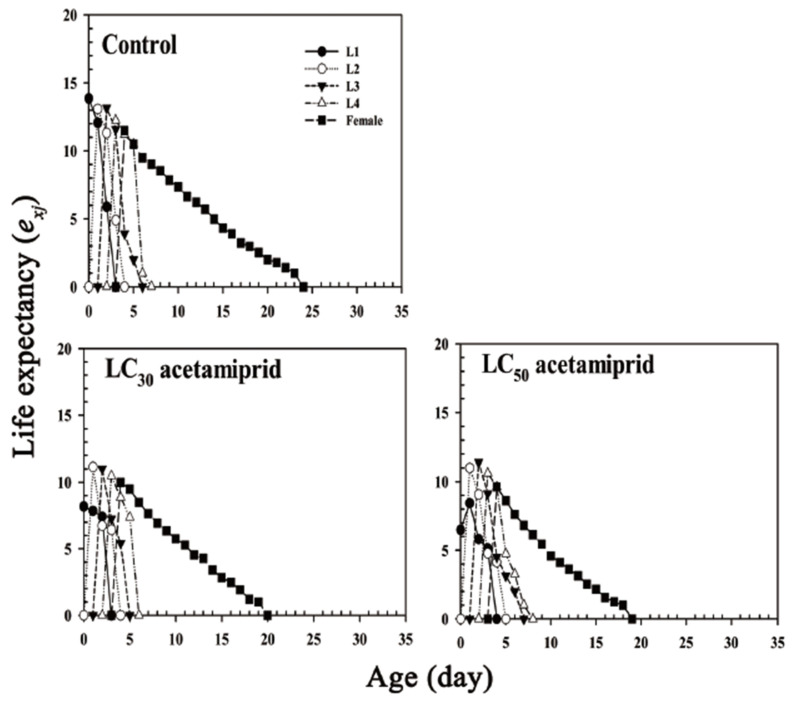
Life expectancy (*e_xj_*) of *Aphis glycines*.

The age-stage-specific life expectancy (*e_xj_*) is the duration that an individual of age *x* and stage *j* is expected to survive after age *x* (Figure 4). The *e_xj_* curves indicated that the life expectancy of adults exposed to acetamiprid once was shorter than that of the control. The highest peak values of life expectancy (*e_xj_*) of the 1st to 4th instar nymphs and adults in the control groups were higher than those of individuals in the acetamiprid treatment groups (Figure 4).

The *e_xj_* of *Aphis* *glycines* were exposed to the following treatments: control, acetamiprid LC_30_, and acetamiprid LC_50_. L1, L2, L3, and L4 represent *e_xj_* of the first, second, third, and fourth instar nymphs, respectively.

## 4. Discussion

In the present study, acetamiprid at the LC_30_ and LC_50_ concentrations had different effects on the reproductive parameters of soybean aphids. Acetamiprid at LC_50_ significantly extended the APOP and TPOP and significantly decreased the mean fecundity per female adult compared with those of the control; conversely, the LC_30_ shortened the APOP, TPOP, and *T* significantly, compared to those of the control. The reason for this discrepancy could be that acetamiprid at different concentrations caused differences in hormesis in soybean aphids [30]. The differences could be linked to the different response strategies of soybean aphids to LC_30_ and LC_50_ acetamiprid-induced stress [31]. Although acetamiprid at LC_50_ and LC_30_ reduced the biological fitness of soybean aphids in the F0 generation, the survival rate, longevity, and fecundity of soybean aphids were lower under LC_50_ than under LC_30_. Exposure to acetamiprid at LC_50_ could lead to direct death of individuals or the retardation of development and reproduction in the surviving individuals. The stress caused by LC_30_ acetamiprid was weaker than that by LC_50_ acetamiprid, and the pressure of acetamiprid at the low lethal concentration on aphids could have induced gradual adaptation to the stress. Soybean aphids might adapt to stress by enhancing their detoxification mechanism to accelerate the acetamiprid metabolism [10,32]. For instance, the upregulation of the expression of certain P450 genes was observed to have accelerated acetamiprid metabolism in a resistant population of *A. gossypii* [10]. In addition, soybean aphids entered the reproduction stage earlier by shortening the generation time and rapidly producing more progeny to facilitate the continuation of the population under stressed conditions [32]. These findings were consistent with the finding that both F1 pre-adult and pre-reproductive periods were shortened when *Myzus persicae* Sulzer (Hemiptera: Aphididae) was treated with LC_25_ flupyradifurone [33].

The effect of acetamiprid at different concentrations on the reproduction of soybean aphids was also manifested in a change in the reproductive peak period of adults. Individuals in the LC_50_ acetamiprid group reached their reproductive peak 1 day later than those in the control group. By contrast, the individuals in the LC_30_ acetamiprid group reached their reproductive peak 2 days earlier than those in the control group. Such trends could be attributed to the different biological and ecological effects of acetamiprid at different concentrations on aphids [32]. They may also be as a result of the delays caused to the development of the ovary by acetamiprid at high concentrations, whereas at low concentrations, acetamiprid could accelerate ovary maturation. For example, the LC_25_ of triazophos was reported to have promoted the significant expressions of *SfVg*, *SfVg*-like, and *SfVgR* genes in the white-backed planthopper, *Sogatella furcifera* Horvath. This pesticide increased the Vg content, an important protein that promotes ovary development, and promoted the mass reproduction of white-backed planthoppers [34].

Pest populations often recover after the frequent application of pesticides in the field, which might be caused by transgenerational hormesis in which low or sublethal concentrations of pesticides stimulate population reproduction [7,35]. Transgenerational hormesis might be a manifestation of the gradual adaptation of soybean aphids to insecticide stress [31]. For example, Rix et al. demonstrated that prolonged exposure to sublethal imidacloprid concentrations significantly increased the reproduction of multi-generational *M. persicae* [32]. Tang et al. also found that the mean generation time of *M. persicae* in the F2 generation was shorter than that in the F1 generation when they were treated with LC_25_ flupyradifurone [33]. Transgenerational reproductive stimulation following exposure to low or sublethal concentrations of pesticides might be due to the transfer of neonicotinoids at low concentrations from parents that survived after pesticide exposure to progeny [36]. Transgenerational effects that stimulate reproduction in progeny might lead to the subsequent production of more fertile progeny by surviving individuals. The amplification of rapid reproduction in progeny over successive generations would seriously threaten the production of soybean and other crops, and could lead to premature outbreaks of soybean aphid populations in the field [37]. Therefore, it is necessary to monitor the population dynamics of soybean aphids and determine the type and concentration of insecticides to apply based on their developmental and reproduction characteristics. Future studies should continue to use the life table and other models to study the effects of acetamiprid application on insect–pest physiological indices at different developmental stages, and to provide more data to facilitate the effective acetamiprid control of soybean aphids.

## 5. Conclusions

The results showed that although acetamiprid at LC_30_ and LC_50_ could reduce the biological fitness of soybean aphids, low concentrations of insecticides could shorten the generation time and pre-reproductive periods, thus accelerating the generation of progeny. These results supported the view that insecticides at the low lethal concentration had hormesis effects on pest populations. In addition, this study also found that a low lethal concentration of insecticides could advance the occurrence of the reproductive peak, which laid a foundation for understanding the major occurrence period of soybean aphids under insecticide stress. In the future, we will continue to explore the effects of acetamiprid on the physiology and behavior of soybean aphids after multiple generations of stress. This will provide further reference for the use of neonicotinoids.

## Figures and Tables

**Table 1:** Toxicity of acetamiprid on the first instar nymphs of *Aphis glycines*.

Insecticide	LC_50_ (mg a.i./L)	95% CI	LC_30_ (mg a.i./L)	95% CI	Slope ± SE ^†^	χ^2^ (df)
Acetamiprid	6.742	5.133–8.629	3.968	2.676–5.203	2.278 ± 0.337	0.157 (5)

^†^ SE = Standard error; CI = Confidence interval.

**Table 2:** Mean values (±SE) of the life–history parameters of *Aphis glycines* exposed to acetamiprid.

Stages	Control	LC_30_	LC_50_
	Mean ± SE ^†^	Mean ± SE	Mean ± SE
L1 development time (d)	1.23 ± 0.04 b	1.21 ± 0.06 b	1.62 ± 0.11 a
L2 development time (d)	1.2 ± 0.04 a	1.14 ± 0.05 a	1.28 ± 0.06 a
L3 development time (d)	1.13 ± 0.05 a	1.12 ± 0.04 a	1.22 ± 0.08 a
L4 development time (d)	1.01 ± 0.01 a	1.04 ± 0.03 a	1.08 ± 0.04 a
Mean longevity of female adult (d)	11.02 ± 0.55 a	9.8 ± 0.59 ab	8.89 ± 0.53 b
APOP (d)	0.20 ± 0.04 b	0.02 ± 0.02 c	1.00 ± 0.16 a
TPOP (d)	4.66 ± 0.07 b	4.31 ± 0.08 c	5.74 ± 0.16 a
Fecundity	42.49 ± 1.83 a	27.73 ± 1.88 b	14.76 ± 1.21 c

^†^ SE = Standard error. Means (±SE) followed by different letters in the same row are significantly different when calculated using the paired bootstrap test at the *p* < 0.05 level. L1 = first instar nymphs; L2 = second instar nymphs; L3 = third instar nymphs; L4 = fourth instar nymphs; fecundity = mean fecundity per female adult. APOP, adult pre-reproductive period; TPOP, total pre-reproductive period.

**Table 3:** Mean value (±SE) of fertility parameters of *Aphis glycines* exposed to acetamiprid.

Population Parameters	Control	LC_30_	LC_50_
	Mean ± SE ^†^	Mean ± SE	Mean ± SE
Intrinsic rate of increase (*r*) (d^−1^)	0.4387 ± 0.0080 a	0.3580 ± 0.0171 b	0.1859 ± 0.0171 c
Finite rate of increase (*λ*) (d^−1^)	1.5507 ± 0.0124 a	1.4304 ± 0.0244 b	1.2043 ± 0.0205 c
Net reproductive rate (*R*_0_)	36.54 ± 2.156 a	14.14 ± 1.684 b	5.61 ± 0.847 c
Mean generation time (*T*) (d)	8.20 ± 0.076 b	7.4 ± 0.106 c	9.28 ± 0.111 a

^†^ Means (±SE) followed by different letters in the same row are significantly different when calculated using the paired bootstrap test at the *p* < 0.05 level.

## Data Availability

Not applicable.
